# Priming psychic and conjuring abilities of a magic demonstration influences event interpretation and random number generation biases

**DOI:** 10.3389/fpsyg.2014.01542

**Published:** 2015-01-21

**Authors:** Christine Mohr, Nikolaos Koutrakis, Gustav Kuhn

**Affiliations:** ^1^Institute of Psychology, University of LausanneLausanne, Switzerland; ^2^Department of Psychology, Goldsmiths – University of LondonLondon, UK

**Keywords:** magical beliefs, magical thinking, magic, paranormal beliefs, belief formation, cognitive biases

## Abstract

Magical ideation and belief in the paranormal is considered to represent a trait-like character; people either believe in it or not. Yet, anecdotes indicate that exposure to an anomalous event can turn skeptics into believers. This transformation is likely to be accompanied by altered cognitive functioning such as impaired judgments of event likelihood. Here, we investigated whether the exposure to an anomalous event changes individuals’ explicit traditional (religious) and non-traditional (e.g., paranormal) beliefs as well as cognitive biases that have previously been associated with non-traditional beliefs, e.g., repetition avoidance when producing random numbers in a mental dice task. In a classroom, 91 students saw a magic demonstration after their psychology lecture. Before the demonstration, half of the students were told that the performance was done respectively by a conjuror (magician group) or a psychic (psychic group). The instruction influenced participants’ explanations of the anomalous event. Participants in the magician, as compared to the psychic group, were more likely to explain the event through conjuring abilities while the reverse was true for psychic abilities. Moreover, these explanations correlated positively with their prior traditional and non-traditional beliefs. Finally, we observed that the psychic group showed more repetition avoidance than the magician group, and this effect remained the same regardless of whether assessed before or after the magic demonstration. We conclude that pre-existing beliefs and contextual suggestions both influence people’s interpretations of anomalous events and associated cognitive biases. Beliefs and associated cognitive biases are likely flexible well into adulthood and change with actual life events.

## INTRODUCTION

Magical thinking refers to a thinking style that “*involves reasoning based on some sort of misconception about, causality, or about natural laws more generally*” (Woolley, 1997 p. 993). Piaget (1927) showed that up to the age of about 12 years, magical thinking forms a major part of children’s inner world (but see Rosengren and Hickling, 1994 for earlier estimates). Despite refinements to this early claim, recent evidence still suggests that children show a more blurred distinction between reality and imagination than adults (Rosengren and Hickling, 1994; Woolley, 1997; Subbotsky, 2010). With increasing age, magical thinking is assumed to dissipate. For example, children from the age of 5 years replace magical explanations increasingly through rational explanations when seeing magic tricks (Rosengren and Hickling, 1994). This developmental perspective goes hand in hand with the views that adults have become rational thinkers shaped through personal, educational, and societal growth (Rosengren and Hickling, 1994).

While these perspectives might be comfortable in our Western, highly educated society, they are not supported by studies investigating magical and paranormal beliefs and experiences in the wider adult population^1^. For instance, only about 10% of the general US population would label themselves as being skeptical toward the paranormal (Rice, 2003). In Europe, 90% of a Swiss sample reported having exceptional experiences (Landolt et al., 2014), and the German public seems pretty open-minded about exceptional experiences, and more than half of the German public report having had such experiences (Knittel and Schetsche, 2012). Moreover, after experiencing anomalous events, Western adults typically deny magical beliefs on an explicit level, but frequently acknowledge implicitly, that an anomalous event had occurred (e.g., turning a drawing into a real object; Subbotsky and Quinteros, 2002; Subbotsky, 2004). Overall, magical beliefs differ widely between individuals of different ages (Rosengren and Hickling, 1994; Subbotsky, 2004) as well as between individuals of the same age (Johnson and Harris, 1994; Subbotsky and Quinteros, 2002). Once these beliefs are established, they seem to be persistent, and factors such as education do surprisingly little to diminish the propensity for these beliefs (Walker et al., 2001; Dougherty, 2004; Genovese, 2005).

Apart from the observation that magical beliefs are common, they seem to go along with specific cognitive biases. For instance, individuals high as compared to low in magical beliefs more frequently see patterns in random noise (Brugger et al., 1993; Blackmore and Moore, 1994), show enhanced illusory face perception (Riekki et al., 2013) or misjudge the probability of events (Brugger et al., 1990; Bressan, 2002). Moreover, believers are more likely to accept bogus personality descriptions (Mason and Budge, 2011), report on events that have never occurred (Tsakanikos and Reed, 2005) and need more time to understand the truth in sentences that violate core knowledge (Lindeman et al., 2008). Such cognitive biases might link with the propensity of magical believers for remote associative processing (Gianotti et al., 2001), fantasy-proneness (Sanchez-Bernardos and Avia, 2006), and openness to experience (Ross et al., 2002). Thus, the literature suggests that magical beliefs are common, highly stable (like trait-like individual differences), and go along with particular cognitive biases and personality variables. Moreover, magical beliefs have likely been established in early childhood. Given this conclusion, it is surprising that relatively little is known about the formation of such beliefs and the causal role of associated cognitive biases.

It is possible that little is known about magical belief formation, at least from adults, because they are considered trait-like, presumably established in early childhood. Yet, there are numerous anecdotal reports that magical thinking can emerge in adulthood, often as a consequence of actual life events. For instance, individuals who experienced near-death-experiences consequentially turned into religious and/or spiritual believers (TrueSpritWorship, 2011, 2013). Freud (1946) reports in one of his Introductory Lectures how his interactions and experiences with patients made him open toward the existence of telepathy and thought-transference. Being initially very critical and skeptical, he changed his opinion following numerous case studies on dreams and the occult. He said “*If one regards oneself as a skeptic, it is as well from time to time to be skeptical about one’s skepticism*” (p. 73). Later on he notes that “*[b]ut I am not concerned to seek anyone’s favor, and I must suggest to you that you should think more kindly of the objective possibility of thought-transference and therefore also of telepathy (…) it seems to me that one is displaying no great trust in science if one cannot rely on it to accept and deal with any occult hypothesis that may turn out to be correct”* (p. 75). These examples illustrate that actual life events can turn formerly skeptical thinkers into magical believers, and that belief formation can occur in adulthood.

In the laboratory, we are aware of a few studies that have investigated the impact of anomalous experiences on individuals’ magical beliefs. For instance, verbal suggestions enhanced the subjective experience of anomalous events in a fake séance room (Wiseman et al., 2003), in a film presenting psychokinetic abilities (Wiseman and Greening, 2005), or the impression of being observed in a supposedly “haunted” room (Bering et al., 2005). Subbotsky (2004) examined whether adults’ causal beliefs are affected by the presentation of anomalous (magical) causal events. When exposed to a magic trick within a magical context (mind-over-matter magic spell), adults were unwilling to accept that the magic action (spell) could have caused the anomalous event. When the anomalous event was not presented within a magical context, but an unrelated event was executed during the anomalous event (e.g., switching a light on and off), adults were prone to causally link the unrelated event with the anomalous event. Thus, while rejecting the possibility of anomalous events explicitly, adults’ implicit behavior showed that the possibility of an anomalous event was nevertheless acknowledged (Subbotsky, 2001).

Most relevant to our study, Benassi et al. (1980) argued that both the public and scientists can be fooled into attributing psychic powers to ordinary and amateur magic routines, and that attributed psychic powers might prevail, even when the performer labels himself as a conjuror. In their study, a magician presented magic tricks in the classroom. The magician was either introduced as a psychic (psychic condition) or a magician (magician condition). After observing the demonstration, participants in the psychic as compared to the magician condition explained the event more strongly via psychic abilities. While this experimental manipulation is promising in showing that framing influences how people interpret an anomalous event, the authors did not assess magical beliefs and reasoning about the event before and after the demonstration. This omission renders causal inferences difficult. Yet, overall this is a promising approach to investigate how actual life events influence our magical beliefs in adulthood.

In sum, the studies above show that experiencing anomalous events can change people’s magical interpretations (and potentially beliefs). These events might also influence cognitive biases that are commonly associated with trait-like magical beliefs. Empirical evidence for such causal claims is still missing. Our aim was to investigate whether the exposure to a magical demonstration, and its contextual presentation (framing), would influence (i) how the event is interpreted (psychic event, conjuring trick, religious miracle, see also Benassi et al., 1980), (ii) self-reported traditional (religious, henceforth TB) and non-traditional (e.g., magical, paranormal, henceforth NTB) beliefs (Tobacky, 2004), and (iii) judgments of event likelihood (repetition avoidance in a random number generation task; Brugger et al., 1990). Former studies have found stronger repetition avoidance in believers in the paranormal compared to skeptics (Brugger et al., 1990), and as the mental number generation task can be performed in a classroom, it was deemed ideal for the current context. In our study, students saw the same magic demonstration and received either the psychic information or the magician information (random allocation, in written format; see also Benassi et al., 1980). As participants saw the same demonstration, but having received different contextual information, we could investigate whether this framing results in more psychic explanations, NTB, and repetition avoidance in the psychic as compared to the magician group.

## MATERIALS AND METHODS

### PARTICIPANTS

The psychology lecture of that day was attended by 91 students (17 male) with a mean age of 20.5 years (SD = 4.12 years). This gender distribution is common in psychology courses. All students were first year Psychology undergraduate students at Goldsmiths – University of London who participated for course credits. The study was approved by the departmental ethics board, and each participant provided written informed consent prior to the experiment.

### SELF-REPORT BELIEF QUESTIONNAIRE

We used the 26-item revised Paranormal Belief Scale from Tobacky (2004). This scale can be divided into seven subscales measuring Traditional Religious Belief, Psi, Witchcraft, Superstition, Spiritualism, Extraordinary Life Forms, and Precognition. The four traditional religious belief items were summed so to represent the TB score, and the remaining items to represent the NTB score. Item examples include “Some psychics can accurately predict the future” (NTB), “Mind reading is not possible” (NTB), and “There is a heaven and hell” (TB). Items are formulated such that participants are asked to answer along a 7-point Likert scale ranging from 1 (strongly disagree) to 7 (strongly agree). Accounting for reverse coded items, the scores are summed so that higher scores reflect greater beliefs. We had no *a priori* prediction that the different NTB subscales would be differentially sensitive to our manipulation. To account for the possibility that TB (or practices) are nevertheless more sensitive to cultural influences than NTB (MacDonald, 1995; Orenstein, 2002) we summed the scores for the TB score (*n* = 4 items) and the remaining items into the NTB score (*n* = 22 items).

### EVENT INTERPRETATION

Benassi et al. (1980) asked participants to write down “comments opinions and reactions about what they had seen,” and then scored this qualitative data according to whether participants thought the performer was a psychic, magician or whether it contained religious-demonic themes. Instead of collecting qualitative data, we asked participants to rate on a 7-point Likert scale ranging from 1 (strongly disagree) to 7 (strongly agree), whether the performance was accomplished through (1) paranormal, psychic or supernatural powers (psychic explanation), (2) ordinary magic trickery (conjuror explanation), or (3) religious miracles (religious explanation). We included the religious miracle measure as it allowed us to compare NB with the extent to which the event was explained using religious explanations. Secondly, Benassi et al. (1980) the only comparable study, asked about religious explanations. Thirdly, it provided us with a control condition (not all beliefs should be endorsed to the same extent).

### MENTAL DICE TASK (BRUGGER ET AL., 1990)

Participants received written and verbal instructions to imagine throwing a dice each time they heard a beep and to write down the number that they imagined being on top of the dice (66 trials). Thus, every second for 66 s, we presented a beep produced by a computer, and the participant was expected to write down a number for each beep. We calculated the repetitions in the number sequence (i.e., 1-1, 2-2, 3-3). If the number generation would be entirely random we would expect participants to produce on average 11 repetitions. Previous research has shown that we avoid repetitions, and that this repetition avoidance is even stronger for individuals with high as compared to low NTB (Brugger et al., 1990).

### PSYCHIC DEMONSTRATION

The psychic demonstration was performed by a professional magician and member of the Magic Circle (http://www.themagiccircle.co.uk). The magician selected a volunteer from the audience. This female volunteer was asked to write down the names of five people who were alive and one deceased person on six pieces of paper. The magician then placed the pieces of paper upside down on the table and placed a lit candle on each of the notes. The magician explained that he was able to use his spiritual powers to contact the dead and asked the volunteer to blow out all of the candles. Approximately 20 s after the candles were blown out, one of them re-ignited and it was the candle that was on top of the piece of paper associated with the deceased person. The candle (i.e., the magician) was correct.

### PROCEDURE

**FIGURE 1:**
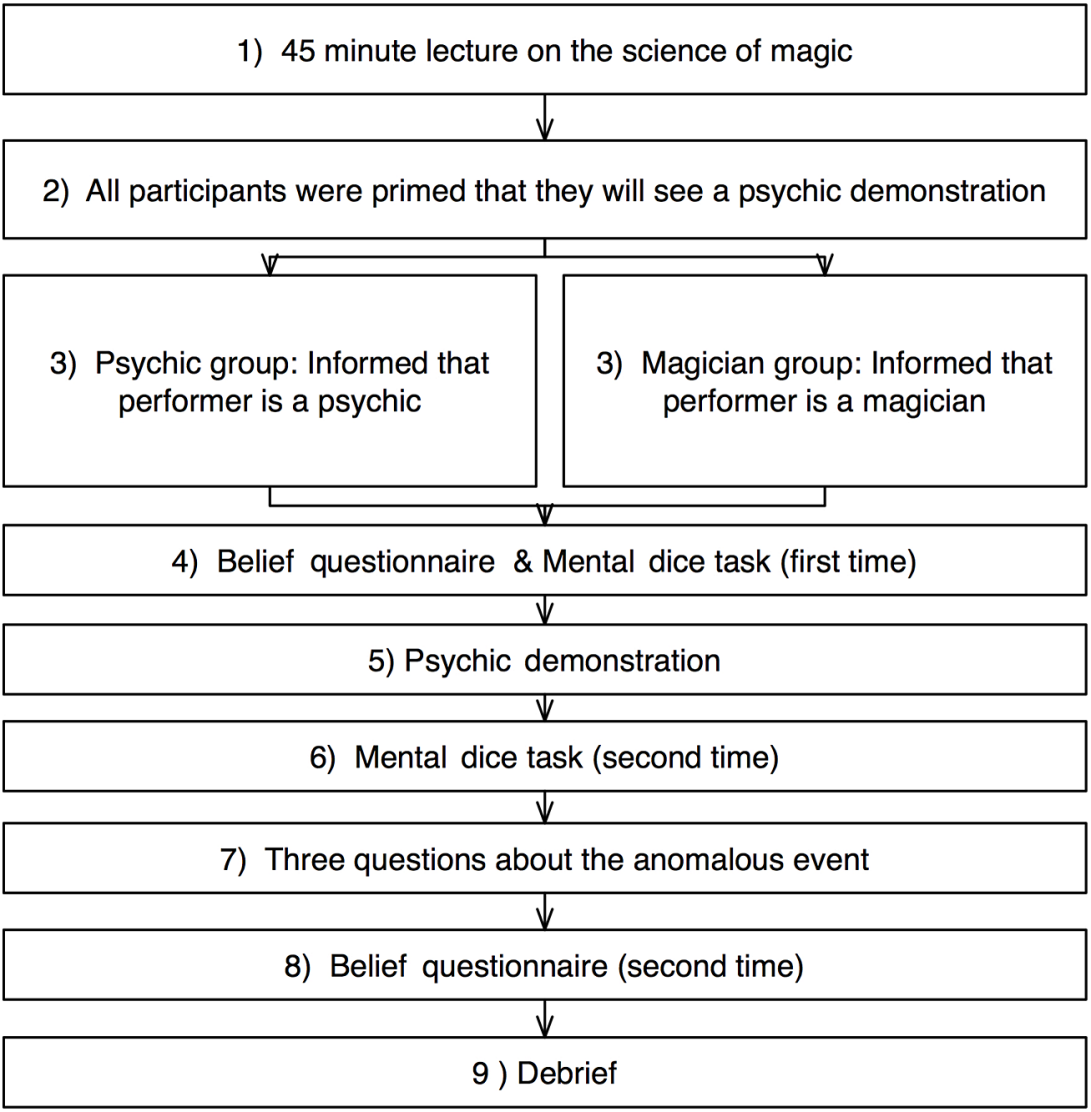
**Schematic demonstration of the sequence of events for participants in the magician and in the psychic groups**.

**Figure 1** shows a diagram of the sequence of events. The experiment was conducted as part of a lecture series on current issues in psychological research and was framed as a demonstration into psychic abilities. In more detail, participants had attended a lecture on the science of magic (given by Gustav Kuhn) prior to the actual experiment (see first event in **Figure 1**). In this lecture, Gustav Kuhn discussed how misdirection can be used to study visual attention. Subsequently, participants were separated by at least one seat and were instructed to refrain from communicating with fellow students throughout the experiment. At this point, all participants were primed to experience a real psychic demonstration (second event in **Figure 1**), i.e., Gustav Kuhn gave them the following verbal briefing. “*As you will be aware, the Anomalistic Psychology Unit at Goldsmith has a keen interest in investigating psychic abilities. Over the years we have carried out numerous experiments to test whether the claims made by psychics hold up on closer scrutiny. Whilst most of the individuals tested so far generally fail these tests, we were very fortunate in that we did find one person who passed most of the preliminarily tests (8/10). His name is Lee and whilst not perfect, his performance was significantly better than chance (p < 0.0032). Lee has told us that he has been developing a presentation of his psychic abilities, and has asked us if he could present it to you and get your opinions and reactions. I thought that this would be very interesting, and so I agreed to let him do it.” [Overall, and in particular the last sentences, instructions were paraphrased from Benassi et al. (1980)]*. After these general instructions, participants were given a work booklet that contained all of the questionnaires and some additional information. Participants were randomly assigned to the magician or psychic condition (third event in **Figure 1**).

Contextual framing instruction for the magic demonstration: the instruction stated that the anomalous demonstration was carried out by a magician pretending to do a fake psychic demonstration, and they read the following statement: “*Some magicians can perform exactly what psychics claim to be doing using ordinary stage trickery.” In fact, Lee is not a real psychic, but a professional magician and member of the Magic Circle. What you are about to see is a demonstration of Lee’s conjuring skills.*

Contextual framing instruction for the psychic demonstration: the instruction stated that the anomalous demonstration was carried out by a true psychic. They read the following statement “*Lee has worked as a Psychic for several years.” Lee is very highly regarded by the European Psychic Society and has astonished numerous well-known scientists by demonstrating his psychic abilities under tightly controlled conditions.*

Immediately afterward, participants filled out the belief questionnaire (Tobacky, 2004; **Figure 1**). Subsequently, they were asked to perform the mental dice task (Brugger et al., 1990; fourth event in **Figure 1**). Once completed, the lecturer introduced the students to the magician who performed the psychic demonstration (fifth event in **Figure 1**). After the demonstration, the students were asked to perform the mental dice task again (Brugger et al., 1990; sixth event in **Figure 1**). Subsequently, they were asked three questions on how they explain the event (seventh event in **Figure 1**): (1) *Whether the performance was accomplished through paranormal, psychic or supernatural powers (psychic explanation), (2) what they have seen has been accomplished by ordinary magic trickery (conjuror explanation), and (3) what they have seen has been accomplished by a religious miracle (religious explanations.* Finally, participants completed the belief questionnaire again (Tobacky, 2004; eighth event in **Figure 1**), before being fully debriefed about the purpose of the experiment (ninth event in **Figure 1**). Here, the magician explained the method behind the effect.

## RESULTS

Five participants provided incomplete data on the mental dice task and were excluded from further analysis.

### RELATIONSHIP BETWEEN CONTEXTUAL FRAMING AND INTERPRETATION OF THE EVENT

To investigate how the two groups interpreted the causes of the anomalous event, we performed a 3×2 ANOVA (analysis of variance) on the explanation ratings with explanation (psychic, conjuror, religious) as within-participant factor and instruction group (psychic, magician) as between-participant factor (**Table 1**). This ANOVA showed a significant main effect of explanation, *F*(2,178) = 163, *p* < 0.00005, η = 0.65. *Post hoc t*-tests indicated that participants provided higher conjuror explanation ratings than psychic and religious explanation ratings, respectively (all *p*s < 0.0005). Moreover, the psychic explanation ratings were higher than the religious explanation ratings (*p* < 0.00005). There was no significant main effect of group, *F*(2,178) = 0.00, *p* = 0.985, η = 0.000, but a significant group by explanation interaction, *F*(2,178) = 6.35, *p* = 0.002, η = 0.067. Participants in the psychic group gave higher psychic explanation ratings than participants in the magician group, *t*(89) = 2.04, *p* = 0.044. On the other hand, participants in the magician group gave higher conjuror explanation ratings than participants in the psychic group, *t*(89) = 2.77, *p* = 0.007. There was no significant group difference for the religious explanation ratings, *t*(89) = 0.69, *p* = 0.50 (**Table 1**). Thus, the contextual framing influenced participants’ psychic and conjuring explanations, but not religious explanations, which were low for both groups (**Table 1**).

**Table 1:** Mean psychic, conjuror, and religious explanation ratings ranging from 1 (strongly disagree) to 7 (strongly agree) for the psychic and the magician group separately.

	Psychic explanation		Conjuror explanation		Religious explanation	
	***M***	**SD**	***M***	**SD**	***M***	**SD**
Psychic	2.82	1.85	5.02	1.70	1.64	1.14
Magician	2.11	1.48	5.89	1.27	1.47	1.20

### RELATIONSHIP BETWEEN BASELINE BELIEF AND INTERPRETATION OF THE EVENT

We correlated participants’ belief scores before the anomalous event with the explanation ratings after the anomalous event (psychic, conjuror, religious; **Table 2**). TB and NTB scores were both significantly correlated with the Psychic and Religious explanation ratings (**Table 2**). Thus, the higher individuals’ beliefs, the more likely were psychic and religious explanations (see also Orenstein, 2002). We also observed a significant correlation between NTB scores and conjuring explanation ratings. The more individuals reported NTB, the less likely were conjuring explanations.

**Table 2:** Pearson correlation coefficients when correlating belief scores (NTB, TB), as assessed before the anomalous event, with the three explanation ratings for the event, as assessed after the anomalous event (*p < 0.05; **p < 0.0005).

	NTB scores	TB scores
Psychic explanation	0.48**	0.41**
Conjuring explanation	-0.26*	-0.08
Religious explanation	0.33**	0.41**

### EFFECT OF CONTEXTUAL FRAMING AND ANOMALOUS EVENT ON EXPLICIT BELIEFS

We investigated whether contextual framing and exposure to the anomalous event influenced participants’ TB and NTB as assessed before and after the demonstration. We made the following assumptions. Firstly, we can attribute group differences in belief scores assessed before the demonstration to contextual framing effects. Secondly, we can attribute group differences in belief scores as assessed after the anomalous event to the experience itself combined with the contextual framing.

We subjected the TB and NTB scores to separate ANOVAs with instruction group (psychic, magician) as between-subject factor and time (before, after) as repeated factor. The ANOVA on TB found no significant main effect of group, *F*(1,89) = 0.028, *p* = 0.87, η = 0.000, no main effect of time, *F*(1,89) = 2.15, *p* = 0.15, η = 0.024, and no group by time interaction, *F*(1,89) = 2.15, *p* = 0.15, η = 0.024 (**Table 1**). The ANOVA on NTB showed a marginal, yet non-significant main effect of group, *F*(1,89) = 2.63, *p* = 0.055 (one-tailed), η = 0.029, and no significant time by group interaction, *F*(1,89) = 0.25, *p* = 0.64, η = 0.002. The main effect of time was significant, *F*(1,89) = 5.70, *p* = 0.019, η = 0.060, wherein NTB scores before the anomalous event were higher than the NTB scores after the event (**Table 3**).

**Table 3:** Mean belief scores (TB, NTB) before and after exposure to the anomalous event for the psychic and the magician group separately.

	Traditional religious belief				Non-traditional religious belief			
	Before		After		Before		After	
Group	*M*	SD	*M*	SD	*M*	SD	*M*	SD
Psychic	3.85	2.01	3.85	2.10	2.88	1.13	2.77	1.10
Magician	3.84	2.34	3.71	2.19	2.52	0.91	2.44	0.94

### EFFECT OF CONTEXTUAL FRAMING AND ANOMALOUS EVENTS ON RANDOM NUMBER GENERATION

**FIGURE 2:**
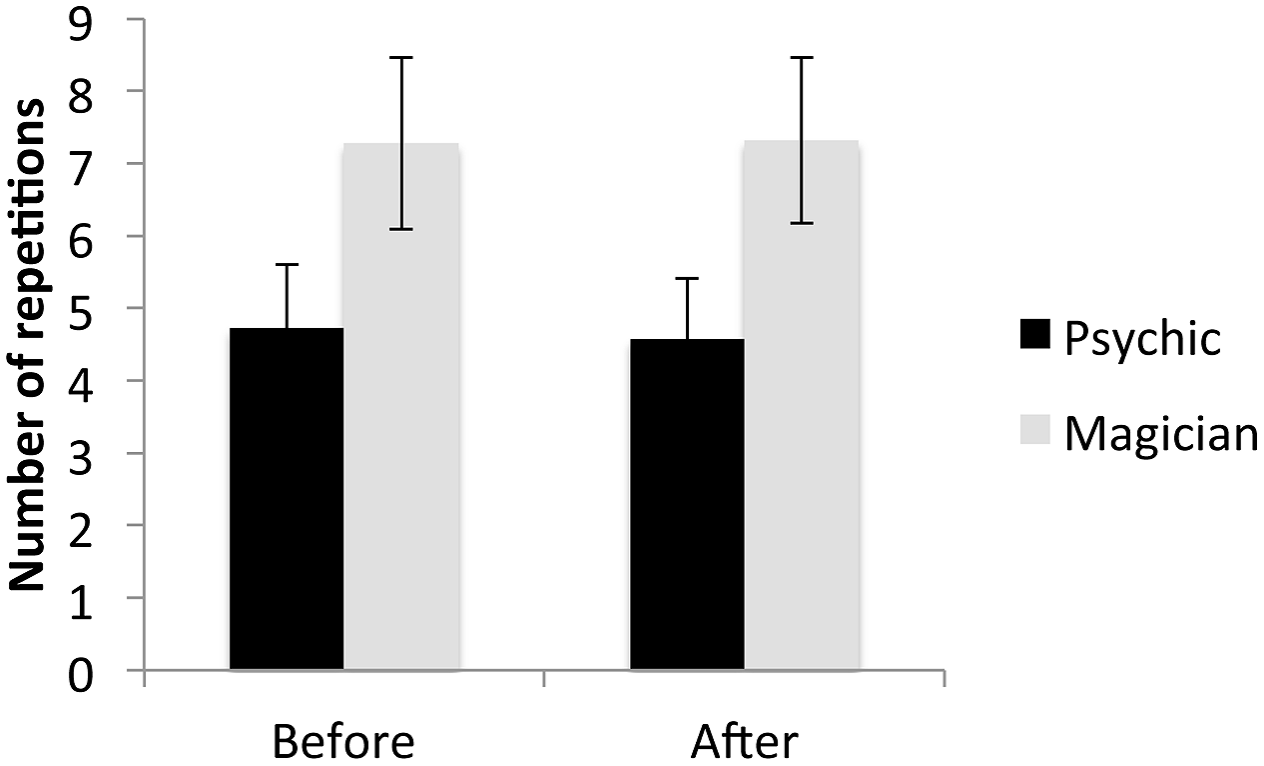
**Mean number of repetitions in the mental dice task before and after the demonstration of the anomalous event as a function of the contextual framing condition (error bars denote standard errors)**.

We performed a 2×2 ANOVA with group (psychic, magician) as between-subject factor and time (before, after) as repeated factor on the number of repetitions. We found a significant main effect of group, *F*(1,89) = 3.74, *p* = 0.028 (one-tailed) η = 0.040, no effect of time *F*(1,89) = 0.015, *p* = 0.90, η = 0.000, and no group by time interaction, *F*(1,89) = 0.046, *p* = 0.83, η = 0.001 (**Figure 2**). The main effect of group emerged from the magician group producing more repetitions than the psychic group. If numbers were generated entirely randomly, we would expect 11 repetitions. As shown in **Figure 2**, participants produced fewer than the expected 11 repetitions. Pearson correlations showed no significant correlations between repetition avoidance (before the anomalous event) and TB scores (*r* = 0.074, *p* = 0.49) and NTB scores (*r* = 0.01, *p* = 0.93).

## DISCUSSION

We investigated whether exposure to an anomalous event changes people’s beliefs and associated cognitive biases (i.e., impaired judgments of event likelihood). Students observed a magic demonstration in a classroom setting and half of the participants were told that the performer was a magician whilst the others were told he is a psychic. Subsequently, participants were asked how they interpreted the demonstration (psychic, conjuring, religious explanations). Participants also filled in a self-report belief questionnaire and performed a random number generation task (mental dice task) before and after the demonstration. Our results showed that (i) participants gave explanations in predicable ways (the psychic group gave more psychic explanations than the magic group; the opposite was true for conjuring explanations; religious explanations were overall low and did not differ between the psychic and the magic group), (ii) baseline belief scores correlated with explanation ratings (higher TB and NTB scores correlated with psychic explanations, higher TB scores correlated with more religious explanations, and higher NTB scores but not TB scores correlated with less conjuring explanations), (iii) the anomalous demonstration had little influence on self-reported beliefs (NTB were lower after as compared to before the demonstration), and (iv) individuals in the psychic group showed stronger repetition avoidance than individuals in the magician group.

We will first discuss the role of contextual framing on our dependent measures, (i.e., the NTB scores and repetition avoidance), because the exposure to anomalous events seemed to have little influence on peoples’ NTB and associated cognitive biases. It is possible that exposure to anomalous events has no impact on NTB and repetition avoidance. While counter to our predictions, this conclusion would support the notion that NTB are well-established in adulthood and show little change, not even with scientific education (Walker et al., 2001; Dougherty, 2004; Genovese, 2005). Before accepting that NTB and associated cognitive biases are fixed and do not change with experience and context, we conjecture alternative explanations that could account for what we observed.

Firstly, the explanation ratings after the anomalous demonstration indicate that the contextual framing influenced people’s experience of the event, or at least their verbal reflections. When the event was framed as a psychic demonstration, participants gave more psychic explanations than when it was framed as a magic demonstration. The reverse was found for conjuring explanations. These results coincide with those reported by Benassi et al. (1980), who similarly, showed that contextually framing a psychic demonstration influenced subsequent event explanations. These observations are supported by independent studies. For instance, verbal suggestions enhanced the subjective experience of anomalous events in a fake séance room (Wiseman et al., 2003), in a film presenting psychokinetic abilities (Wiseman and Greening, 2005), or in a supposedly “haunted” room (Bering et al., 2005). Subbotsky (2001, 2004) also showed that seemingly skeptical adults demonstrate behavior that implicitly indicates the possibility of anomalous explanations. Moreover, when given hints to explain anomalous events through illusory correlations, many of these seemingly skeptical adults appreciated explanations suggested by such correlations. Whilst the effect of framing did not result in significantly different NTB scores, the trend was certainly in the predicted direction, and our experimental design may have simply lacked sensitivity in picking up these differences (see also limitation section).

Secondly, the results from the mental dice task indicate that the contextual framing was effective. Contextual framing influenced a cognitive bias that has previously been associated with trait-like magical beliefs, i.e., repetition avoidance in a random number generation task (Brugger et al., 1990). More precisely, participants in the psychic group showed a higher level of repetition avoidance than participants in the magician group. This group difference was found irrespective of whether they had seen the anomalous event or not. Thus, cognitive biases associated with beliefs are probably not stable cognitive biases but are influenced by the contextual information and situation. Admittedly, given our initial hypothesis, we predicted that the difference would be particularly apparent in the psychic group after rather than before the anomalous event demonstration. Yet, the demonstration itself did not result in any change in belief scores or cognitive measures, indicating that these measures seem too well-established to change with the one-off anomalous experience. The one-off contextual framing event, on the other hand, was sufficiently powerful to transiently change individuals’ perception and appreciation of the event (Benassi et al., 1980; Bering et al., 2005; Wiseman and Greening, 2005). Presumably, the contextual framing event might be so powerful that the subsequent anomalous experience had no additional impact on the dependent measures. Alternatively, the actual anomalous experience may have been too simple to exert any measurable effects. Future studies should test these possibilities. Particular suggestions and reflections on the powerfulness of the anomalous event demonstration are detailed in the limitation section.

A final observation worth discussing is the drop in NTB scores after the anomalous event demonstration. We assume that this drop in NTB scores reflects a psychometric artifact resulting from a repetition bias or response bias, rather than the anomalous event itself. Previous studies showed that magical ideation was relatively unstable over a 2 years period (Meyer and Hautzinger, 1999) and that magical ideation was lower in a group that had received the contextual information that the questionnaire associates with psychosis as compared to a group that had received the contextual information that the questionnaire associates with creativity (Mohr and Leonards, 2005).

### LIMITATIONS

If one takes the original hypothesis, we can conclude that the contextual framing was a powerful manipulation while the anomalous event demonstration was not. In comparison to Benassi et al. (1980) our participants were generally far more skeptical about the anomalous event. Benassi et al. (1980) asked participants to write down comments, opinions, and reactions about what they had seen. These comments were later scored according to whether the individual indicated that he/she thought that the performer was a psychic or a magician. It is impossible to directly compare this qualitative data with our own, but the fact that 77% of their participants in the psychic condition came up with psychic explanations illustrates that the majority of participants attributed the anomalous event to a psychic cause. This is in stark contrast to our own data, where on average participants “slightly” to “moderately” disagreed with the idea that the anomalous event was accomplished through psychic powers. It is likely that our magic demonstration might have been less striking, and by inference less influential on beliefs and cognitive biases, than the contextual framing manipulation. For instance, Benassi et al. (1980) used a whole range of psychic demonstrations (mindreading, teleportation, metal bending). We, on the other hand, used a simple magic trick that could (with some training) be performed by novice magicians. Thus, future magic demonstrations should include several tricks and extend the demonstration in duration. Moreover, we tested participants in a classroom subsequent to a psychology lecture on the science of magic. Thus, these students were fully aware that the experimenter (Gustav Kuhn) has a keen interest and experience in conjuring. It is likely that our participants were more skeptical about the authenticity of the psychic performance than a naïve audience would have been. Moreover, as our participants were predominantly female, we cannot guarantee that our results generalize to males.

In addition, our participants received the actual contextual framing instructions in written format. We do not know whether they read this instruction properly or not. In Benassi et al. (1980) participants in the two groups were tested at two different occasions receiving the instructions verbally by the experimenter. As it is impossible to guarantee that each performance is identical, we favored the model in which all participants are exposed to the same performance, but participants are given different written instructions. Despite these caveats and methodological differences between studies, we suggest that the overall methodological approach is promising. In particular, despite the simplicity of our magic trick, the classroom setting, having just had a lecture on the science of magic, our participants did not fully dismiss a psychic explanation.

For future studies, we also suggest to consider the context in which an anomalous event is performed. For example, a spiritual reading carried out in a real séance room is likely to be more powerful than when the same demonstration is presented in a classroom context (Wiseman et al., 2003). Moreover, true séances are typically carried out by people with a very strong conviction in the phenomena (Wiseman et al., 2003), something our magician somewhat lacked. Another concern is the repeated use of the belief questionnaire in short succession. Ideally, participants would receive different belief questionnaires that are yet comparable in what they measure, or even better, a well-established belief questionnaire would be split into two comparable halves so that the first half could be provided prior to the presentation and the other half subsequent to the presentation. Due to the comparability of the two halves, the change in scores could be assessed directly. Finally, we might find stronger effects for non-student populations as suggested by the findings of Bressan (2002). Her findings indicated that links between impaired probability judgments and paranormal beliefs are less pronounced in students than in regular workers of varying education.

We outline another concern not covered extensively so far. Benassi et al. (1980) performed a between-subject design in which participants in the psychic group were tested at a different occasion to those tested in the magician group. The formulation of the contextual framing was matched for the first part of the instruction, but differed later between conditions. The magician aimed to perform the demonstration comparably across the different testing sessions. In our study, we preferred to make sure that each participant saw exactly the same performance so that possible performance differences or audience effects would not differ between the magician and psychic group. We formulated the instructions such that they would be suggestive but be free of personal opinion. Indeed, in Benassi et al. (1980) some instructions included a personal judgment of the experimenter. The verbatim instruction in the psychic instruction included for example “I thought that would be interesting, even though I’m not convinced personally of Craig’s or anyone else’s psychic abilities, so I agreed to let him do it” (p. 3). In the strong magic condition, the experimenter added “In his act, Craig will pretend to read minds and demonstrate psychic abilities; but Craig does not really have psychic abilities, and what you’ll be seeing are really only tricks” (p. 3). We do not know to what extent such different formulations add to the observed results by enhancing or attenuating possible effects. However, the careful matching of verbal instruction is advisable.

## CONCLUSION

The present study investigated whether the exposure to an anomalous event would result in a change in NTB and associated cognitive biases. We take the current findings as promising evidence that exposure to an anomalous event (or its announcement) can influence participants’ evaluation of the event together with associated cognitive biases. We conclude that such findings are key to showing that magical beliefs and associated cognitive biases are flexible, not necessarily trait-like, and that this flexibility is possible well into adulthood. We discuss the necessity to further evaluate which types of demonstrations are powerful to lead to belief change if not belief formation. In any case, the current paradigm is promising in showing causal (rather than correlational) factors in belief change, belief formation and the role of associated cognitive biases in these processes.

## Conflict of Interest Statement

The authors declare that the research was conducted in the absence of any commercial or financial relationships that could be construed as a potential conflict of interest.

## Footnotes

1 Based on a recent review, we will treat magical, paranormal, superstitious, and supernatural beliefs interchangeable (Lindeman and Svedholm, 2012).
